# Small-Fiber Neuropathy: An Etiology-Oriented Review

**DOI:** 10.3390/brainsci15020158

**Published:** 2025-02-06

**Authors:** Alessandro Furia, Rocco Liguori, Vincenzo Donadio

**Affiliations:** 1 Dipartimento di Scienze Biomediche e Neuromotorie, University of Bologna, 40138 Bologna, Italy; 2 IRCCS Istituto delle Scienze Neurologiche Di Bologna, UOC Clinica Neurologica, 40139 Bologna, Italy

**Keywords:** small-fiber neuropathy, etiology, skin biopsy, QST, confocal corneal microscopy, evoked potentials

## Abstract

Background: Small-fiber neuropathy (SFN), affecting Aδ or C nerve fibers, is characterized by alterations of pain and temperature sensation, as well as autonomic dysfunction. Its diagnosis may still remain challenging as methods specifically assessing small nerve fibers are not always readily available, and standard techniques for large-fiber neuropathies, such as electroneuromyography, yield negative results. Still, skin biopsy for epidermal innervation and quantitative sensory testing allow for diagnosis in the presence of a congruent clinical picture. Objectives: Many different etiologies may underlie small-fiber neuropathy, of which metabolic (diabetes mellitus/impaired glucose tolerance) and idiopathic remain prevalent. The aim of this narrative review is to provide a general picture of SFN while focusing on the different etiologies described in the literature in order to raise awareness of the variegated set of different causes of SFN and promote adequate diagnostic investigation. Methods: The term “Small-Fiber Neuropathy” was searched on the PubMed database with its different recognized etiologies: the abstracts of the articles were reviewed and described in the article if relevant for a total of 40 studies. Results: Many different disorders have been associated with SFN, even though often in the form of case reports or small case series. Conclusions: Idiopathic forms of SFN remain the most prevalent in the literature, but association with different disorders (e.g., infectious, autoimmune) should prompt investigation for SFN in the presence of a congruent clinical picture (e.g., pain with neuropathic features).

## 1. Introduction

The expression “Small-Fiber Neuropathy” (SFN) defines a wide group of disorders in which nerve fibers of a small caliber are preferentially affected. According to the classification based on dimension, small nerve fibers are defined as follows in the table below [1] (Table 1):

Small nerve fibers serve somatic (pain and temperature sensation) and autonomic functions. Thus, their impairment can lead to sensory and/or vegetative symptoms [2]. Pain is often the cardinal sign of SFN, bearing neuropathic features such as paresthesias with a burning quality or “electric shock” or “painful cold”-like sensation; in some cases, such paresthesias can also be tingling, numbness, or “pins and needles”-like. Moreover, tactile allodynia and hyperesthesia are pathological phenomena specific to neuropathic pain. Impairment in thermoception can lead to hypoesthesia to cold and hot temperatures. Pruritus, both spontaneous and contact-evoked, is a sensory symptom mediated by C fibers expressing TRPV1 and TRPA receptors, which also mediate pain. Itch is indeed often present in disorders affecting small fibers and represents an important clue suggesting a neuropathic origin of pain [3].

Sensory symptoms present more frequently in a length-dependent fashion akin to large-fiber polyneuropathy, starting from the distal side of lower limbs and ascending. Still, non-length-dependent processes may occur, albeit less frequently, and may be more difficult to recognize and diagnose [4,5]. SFN can also be focal, affecting single body areas such as the cephalic region; burning mouth syndrome, characterized by chronic painful sensation in the oral area, has been suggested to be related to trigeminal SFN [6].

Autonomic impairment is less frequent but can affect the various domains of this system, such as the cardiovascular (orthostatic intolerance/hypotension), vasomotor (skin flushing), secretomotor (xerostomia, sialorrhea), gastrointestinal (constipation, diarrhea, gastroparesis), genitourinary (urinary retention/incontinence, erectile dysfunction), thermoregulatory (hypo/hyperthermia, heat/cold intolerance), and pupillomotor (myosis/mydriasis) domains [7].

SFN is a relatively recent pathological entity, as the identification and study of small nerve fiber structure and function had been challenging. Indeed, the impossibility of microscopically identifying epidermal fibers due to technical factors led to the theory that the epidermis did not possess nerve fibers, and it was only in the 1990s that intraepidermal small nerve fibers were demonstrated via staining with protein gene product 9.5 [8].

Diagnostic techniques assessing large nerve fiber integrity and function are now long-standing and firmly established (e.g., electroneuromyography, evoked potentials), while methods to assess small nerve fibers, such as skin biopsy and quantitative sensory testing (QST), are less diffuse. Nonetheless, their application has allowed for the creation of diagnostic criteria for SFN [2,7].

Diagnosis of SFN is still hindered by scarce acknowledgment by treating physicians and insufficient access to diagnostic techniques, which remain available only in specialized centers [9]. The aim of this narrative review is to provide a comprehensive picture of SFN while also focusing on the wide set of etiologies underlying SFN in order to promote recognition of SFN in non-specialized settings dealing with disorders associated with this entity.

## 2. Research Methods

The review is structured in different sections (Diagnosis, Etiologies, Treatment, and Conclusions). Due to the focus of the article on etiologies, a search strategy for the works to include has been employed: on the PubMed database, the terms “diabetes”, “metabolic”, “infection”, “autoimmune”, “vaccination”, “toxic”, “drug-induced”, “chemotherapy”, “inherited”, and “idiopathic” were searched with “AND “small-fiber neuropathy”” attached.

Criteria of inclusion were the use of the English language and research and review articles concerning the specific etiology of the search, thus excluding more general reviews. If studies dealt with neuropathies of both large and small nerve fibers, only data concerning pure SFN were valorized. The search was completed in the period between December 2024 and January 2025.

The research yielded 232 results for “diabetes”, 69 for “metabolic”, 44 for “infection”, 109 for “autoimmune”, 37 for “vaccination”, 28 for “toxic”, 3 for “drug-induced, 31 for “chemotherapy”, 33 for “inherited, and 131 for “idiopathic”. Of these, revision by the authors according to the selection criteria led to the identification of 40 articles, whose data are presented in the Section 4.

## 3. Diagnosis

Precise diagnosis of SFN may be hindered by several issues. Patients may present with extensive pain-related symptoms, while objective clinical signs of small fiber impairment may be difficult to evaluate at the bedside without proper attention. Moreover, techniques assessing small fiber function and integrity are not readily available in most centers [9].

Nonetheless, aside from these more practical aspects, the lack of a true gold-standard diagnostic method for SFN is a key issue which has been extensively discussed and has significantly impacted on the development of diagnostic criteria [2,7,10].

Currently, a diagnosis of SFN can be made based on two different sets of criteria [2,11], as highlighted in Table 2.

The Besta criteria were the first to be proposed: as discussed by the authors themselves in a reappraisal study a decade after their introduction, the combinatory approach of having two of three criteria met for diagnosis was devised to address the lack of a gold standard. In the same study, the Besta and NEURODIAB criteria have shown strong agreement when compared [7].

A clinical picture compatible with SFN is a fundamental aspect in both sets of criteria. More specifically, it has been shown that positivity to only diagnostic techniques (i.e., skin biopsy and QST) without a clinical picture, while compatible with an SFN diagnosis, is inferior to the presence of clinical signs and one positive technique. Furthermore, objective signs and not only subjective symptoms must be taken into account when defining a suggestive clinical picture, as symptoms alone are often not powerful enough to allow for diagnosis and can be unsubstantiated by techniques [7].

As discussed more extensively below, evaluation of autonomic impairment is often more difficult and, while still not incorporated, addition of diagnostic techniques for the autonomic nervous system in diagnostic criteria has been advocated [10].

### 3.1. History and Clinical Examination

Diagnosis of SFN requires a thorough process in which precise anamnestic data must be collected and a detailed clinical examination must be performed, also encompassing evaluation of sensory modalities of small nerve fibers [7].

Family history should be first collected with particular focus on members affected by pain or SFN-related phenomena such as erythromelalgia, suggesting a possible genetic cause of the disorder. Social and occupational history should determine exposure to offending agents such as alcohol. Past medical history should uncover previous exposure to neurotoxic drugs, such as chemotherapy, as well as disclosing disorders commonly associated with SFN, such as diabetes and glycemic intolerance or autoimmune disorders. Present history should then determine how the disorder presented and how it evolved. Care should be taken in exploring autonomic involvement via specific questions encompassing all domains [12].

Questionnaires to evaluate both pain and autonomic symptoms can be administered, including the “Douleur Neuropathique 4” (DN4) for the screening of neuropathic pain [13], the Small-Fiber Neuropathy—Symptom Inventory Questionnaire” (SFN-SIQ) [14] or the Composite Autonomic Symptom Score 31 (COMPASS-31) [15].

Clinical examination should first exclude the involvement of large nerve fibers, thus determining that no motor deficits and no impairment of sensation via the dorsal lemniscus system (e.g., vibration, position sense) are affected. Deep tendon reflexes should not be affected. Physical inspection of the patient may additionally provide valuable information about autonomic dysfunction (e.g., skin trophism and color) [16].

Small fiber function can be first assessed at bedside or in the outpatient setting by employing instruments assessing pain and temperature sensation, such as sterile pins or hot and cold objects. With enough patient cooperation, the area of sensory impairment can be mapped reliably. Specific phenomena such as tactile and mechanical allodynia can be assessed in affected areas [9].

### 3.2. Diagnostic Techniques

The following techniques are employed in clinical and research settings in order to support the diagnosis of SFN or study its pathogenetic mechanisms.

#### 3.2.1. Skin Biopsy

Skin biopsy allows for the quantitative and qualitative evaluation of intraepidermal nerve fibers employing identification via protein gene product 9.5 (PGP 9.5) staining.

Subsequently, fibers are counted, and intraepidermal nerve fiber density (IENFD, i.e., fibers/mm) is calculated [17].

Two techniques of fiber staining are most commonly employed [18]: bright-field immunohistochemistry and indirect immunofluorescence. Different reference values for IENFD have been provided for both techniques [19,20], also adjusting for age and gender, although the exact cutoffs to define SFN have been debated [20].

**Figure 1:**
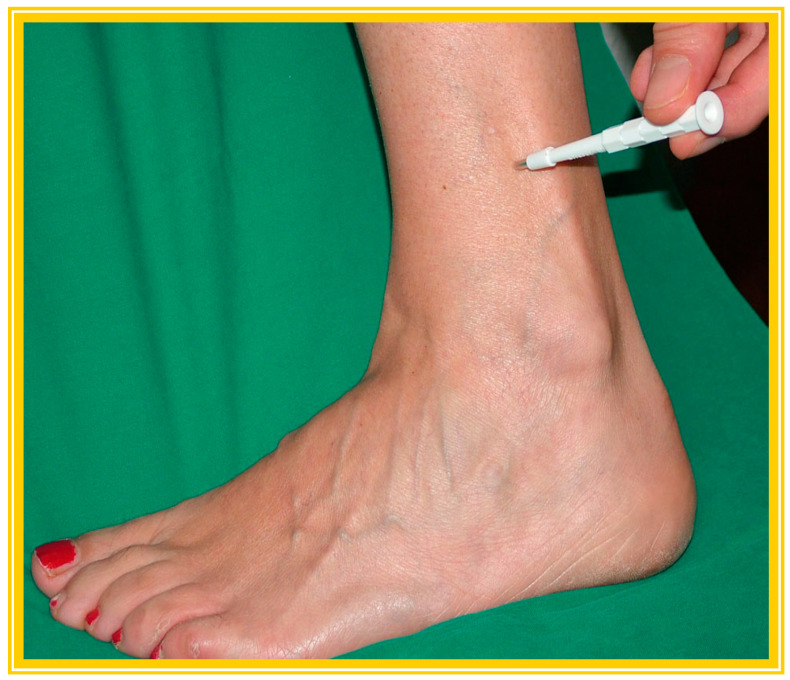
Skin biopsy sampling at lower leg with 3 mm punch.

As shown in Figure 1, biopsy is most commonly performed at the lower limb, at a distal (leg, near malleoli) site.

Samples can also be collected at proximal (thigh, near the knee) sites, thus also allowing for the evaluation of the gradient of fiber loss. While reduced IENFD is the key required finding in SFN diagnosis, structural alterations of nerve fibers have long been appreciated in neuropathies, as well; abnormalities such as axonal swelling and increased branching are thought to suggest an early stage of nerve pathology, which can evolve into full-fledged neuropathy [21,22].

The autonomic nervous system, both with its sympathetic and parasympathetic branches, also innervates skin structures such as blood vessels, sweat glands, and the arrector pilorum muscle. Adrenergic and cholinergic nerve fibers make for a complex innervation picture that has not still been fully elucidated. As highlighted in a recent study [23], sympathetic adrenergic and cholinergic fibers are abundant in skin, while parasympathetic cholinergic fibers can also be found. Most importantly, these fibers express different sets of markers, which allow for the identification of adrenergic (e.g., tyrosine hydroxylase—TH—and dopamine beta-hydroxylase—DbH) and cholinergic (vesicular acetylcholine transporter—VACHT) function. While possible, autonomic profiling is still technically demanding and time-consuming; therefore, obtaining quantitative data from skin biopsy has still not become routine [23]. A limitation of skin biopsy in SFN is that it cannot assess the in vivo function of small fibers, which can be dysregulated in some forms of neuropathy, such as in the case of toxic SFN, in which noxious agents may interfere with ion channel function without altering fiber structure [24].

#### 3.2.2. Quantitative Sensory Testing (QST)

Quantitative sensory testing (QST) refers to batteries of psychophysical testing using instruments specific for different sensory modalities with the aim of determining the sensory thresholds for such modalities. Thus, both large and small nerve fiber functions can be assessed, and a complete sensory profile can be provided [25].

The German Research Network on Neuropathic Pain (DFNS) has devised a standardized protocol with reference values, comprising 7 tests and 13 parameters, comprising cold and warm sensation, thermal pain thresholds, mechanical pain sensitivity, pinprick sensitivity, mechanical allodynia, and pain summation to repetitive pinprick stimuli [26]. The sensory threshold can be determined through different means: in the limits method, a continuous stimulus increasing or decreasing at a fixed rate is administered, and the patient signals when the specific sensation is felt; in the levels method, the stimulus is presented at a fixed value and time period, and the patient indicates whether it was felt, then leading to increases or decreases in the stimulus until a sensation threshold can be determined [25].

QST has been employed in studying SFN caused by different etiologies, such as diabetic [27] and Fabry disease [28], in which this technique determined a particularly altered cold sensation.

While theoretically providing quantitative data on sensory pathway function, QST still relies on full patient cooperation and the comprehension of testing.

#### 3.2.3. Laser-Evoked Potentials

This subgroup of evoked potentials employs painful stimuli that elicit signals from the nociceptive pathways, thus also allowing for the study of small nerve function. Among these are laser-evoked potentials (LEPs) and contact-heat-evoked potentials (CHEPs) [29].

LEPs assess the status of the spinothalamic pathway through the administration of painful stimuli through infrared lasers. LEPs stand out as one of the most reliable techniques for the assessment of nociception, although the recording of C fibers is difficult and not possible from arms, allowing objective and reproducible evaluation of both the peripheral and central pain pathways [30].

#### 3.2.4. Autonomic Testing

These include a range of different tests assessing the function of various autonomic domains. Cardiovascular testing evaluates how the autonomic nervous system exerts control over cardiovascular reflexes. Among such tests is tilt testing, in which the patient is strapped on a bed that is tilted to assess the baroreceptor reflex, and the Valsalva maneuver assessing the cardiovascular response when transient intra-abdominal pressure is applied. Such evaluation is particularly relevant when the patient refers symptoms that could be correlated to orthostatic intolerance (e.g., dizziness when standing up), which could be explained by orthostatic hypotension or tachycardia, as seen in postural orthostatic tachycardia syndrome (POTS) [31].

Other autonomic tests include sudomotor evaluation, which can employ different techniques such as the sympathetic skin response (SSR) and the quantitative sudomotor axon reflex testing (QSART). The SSR uses electrodes on the surface of hands and feet to record a low-frequency response to stimulation (electrical or via other means). The QSART evaluates instead the effect of acetylcholine iontophoresis into the skin, which causes both a direct sudomotor response of the specific area and an indirect response of neighboring regions [29].

#### 3.2.5. Corneal Confocal Microscopy

The cornea is a densely innervated part of the eye, and its nerve fibers play a key role in protective mechanisms, such as tear secretion and the blink reflex [32].

Originally applied in corneal disorders, confocal microscopy of the cornea allows for quantitative and qualitative measurement of innervation. Its first application in the neurological field was in patients with diabetic polyneuropathy, where reduction in innervation preceded symptoms of impaired corneal sensitivity [33]. As highlighted in a review by Petropoulos et al. [34], corneal confocal microscopy has then been applied in the study of both large and small-fiber neuropathy from a wide range of causes, consistently showing abnormalities. Similarly to skin biopsy, altered innervation was also found in neurodegenerative disorders, such as amyotrophic lateral sclerosis [35] and stroke [36]. Thus, with the development of reference values, such a technique represents a promising non-invasive mean of both diagnostic and prognostic values in painful neuropathies, also comprising SFN.

#### 3.2.6. Microneurography

Microneurography allows direct, in vivo characterization of unmyelinated, small fiber activity from peripheral nerves (typically peroneal) by the insertion of a tungsten needle into nerve fascicles. This technique has been widely employed for recording sympathetic outflow activity (either as skin sympathetic nerve activity—MSNA—or muscle sympathetic nerve activity—SSNA) both in physiological and pathological states, such as in patients presenting with dysautonomia [37]. Moreover, microneurographic recording of nociceptor activity from C fibers has also been a longstanding line of research [38]. In particular, microneurography has provided insight into pain-related phenomena such as hyperalgesia [39]. Thus, while not readily applicable to clinical practice, it represents a key technique for research applications.

## 4. Etiology

While SFN has been associated with a multitude of different disorders and pathological processes, it is estimated that 50% of cases remain idiopathic [9].

### 4.1. Metabolic

Diabetes mellitus, either type 1 or 2, is among the most widespread disorders in the world, causing significant morbidity and mortality due to multisystem involvement. This also includes the peripheral nervous system, as diabetes is associated with the development of different types of neuropathies. While length-dependent large-fiber polyneuropathy is most commonly encountered, isolated SFN is possible and thought to represent a precursor form of neuropathy ultimately leading to large-fiber neuropathy; autonomic involvement is also prominent in diabetic SFN [40].

While not fully elucidated, the pathophysiology of diabetic neuropathy is closely related to multisystem derangement induced by hyperglycemia, which, aside from oxidative stress, is at the base of multiple pathogenetic mechanisms, such as alterations of polyol or hexosamine metabolic pathways [41].

Even stages of prediabetes, characterized by impaired fasting glucose or impaired glucose tolerance, are associated with SFN [40].

Metabolic syndromes, particularly hypertriglyceridemia, have been associated with SFN [42].

Hypothyroidism may cause pain syndromes compatible with SFN, as determined by QST sensory thresholds [43].

Vitamin B12 deficiency is more commonly associated with the development of subacute combined degeneration of the spinal cord; however, a large cohort study has found its incidence to be higher in SFN patients when compared to the general population [44].

### 4.2. Infectious

Many infectious agents have been associated with SFN. Infection can cause direct involvement of the nervous system, as is the case for human immunodeficiency or herpes zoster viruses, or they can induce damage via other, multisystemic pathogenetic mechanisms, as is the case for hepatitis viruses leading to nerve damage via both vasculitic and metabolic impairment caused by hepatic impairment [45].

HIV infection, which causes diffuse involvement of both the central and peripheral nervous system, may cause sensory neuropathies with prominent pain, which has been shown to be related to IENFD loss [46]. Among the various hepatitis viruses, hepatitis C infection has been most commonly associated with the development of SFN [47]. Additionally, a case-control study has reported that neuropathic pain was a common complaint of patients with acute hepatitis E infection [48].

Lyme disease can lead to a chronic phase after treatment characterized by cognitive impairment and pain (post-treatment Lyme disease syndrome, PTLDS), which has been related to both somatic and autonomic SFN [49].

Herpes zoster is a disorder caused by reactivation of the varicella zoster virus, which can cause significant neuropathic pain after remission, known as post-herpetic neuralgia. Such a clinical picture has been shown to be caused by SFN [50]. Interestingly, pruritus is a commonly associated feature of herpes zoster. Among other herpes infections, Epstein–Barr virus (EBV) infection was described in a case of acute autonomic neuropathy [51].

Leprosy can cause different types of neuropathies, including clinical pictures dominated by neuropathic pain due to small-fiber neuropathy [52].

COVID infection can lead to a wide array of signs and symptoms in the chronic course (post-COVID), among which painful syndromes have been shown to be partly related to SFN [53,54].

Other agents described in case reports include hantavirus and Angiostrongylus cantonensis [55,56].

### 4.3. Immune-Mediated

Immune-mediated forms of SFN can be associated with immune activation in the context of autoimmune systemic or nervous system-specific disorders. Both large- and small-fiber neuropathy are possible in autoimmune disorders; painful syndromes can also be caused by autoimmune damage of dorsal root ganglia, which further complicates the clinical picture by causing non-length-dependent symptoms [57].

#### 4.3.1. Autoimmune Systemic Disorders

Systemic lupus erythematosus (SLE) is a multisystem autoimmune disease that can involve both the central (e.g., neuropsychiatric symptoms) and peripheral nervous system. SFN has been demonstrated in SLE patients presenting with neuropathic pain. Most interestingly, both length-dependent and non-length-dependent forms of SFN have been observed, the latter postulated to be due to dorsal root ganglion pathology [57].

Sjögren syndrome (SS), another multisystem disorder with a predilection for glandular tissue, has long been associated with the development of sensory neuropathies, typically large-fiber and axonal. However, pure SFN is possible and has been suggested to be associated with a distinct immunological profile [58].

In sarcoidosis, a granulomatous disorder, SFN has been estimated to be the most common neurological complication, associated with autonomic dysfunction [59].

#### 4.3.2. Antibodies in SFN

As highlighted in a recent review [60], autoantibodies have been associated with functional small nerve fiber dysfunction and neuropathic pain and, in some cases, with SFN.

These include antibodies against trisulfated heparin disaccharide (TS-HDS) and fibroblast growth factor receptor-3 (FGFR-3), which have been associated with non-length-dependent SFN. Further research is needed to fully elucidate their role in the development of pain and nerve pathology [60].

### 4.4. Drug-Induced and Toxic

Chemotherapy-induced peripheral neurotoxicity (CIPN) is a known and possibly severe side effect with significant morbidity [61].

Platinum compounds are typical drugs causing CIPN, among which oxaliplatin stands out in that it causes acute cold-induced paresthesias, which have been related to its effect on Na^+^ channels [62].

Bortezomib, used in the treatment of multiple myeloma, has been associated with somatic and autonomic length-dependent SFN [63].

Taxanes have also been shown to cause sensory neuropathy, only affecting small nerve fibers in some cases. More particularly, docetaxel can induce SFN, as demonstrated by skin biopsy and QST [64].

Metronidazole, an antibiotic, can induce both central and peripheral neurotoxic effects, also leading to painful SFN. Nitrofurantoin has shown similar effects on small nerve fibers [65].

Toxic agents such as alcohol cause both large- or predominantly small-fiber neuropathy [66].

### 4.5. Genetic

Genetic causes include syndromes in which SFN is the main presenting picture and inherited systemic disorders in which small fibers can be affected.

These disorders were recently reviewed [67]: in the former group are mutations of genes involving sodium ion channels, including SCN9A, SCN10A, and SCN11A. Pure SFN has been described in hereditary sensory and autonomic neuropathy types 1, 3, and 5 (HSAN1, HSAN3, HSAN5).

SCN9A encodes the voltage-gated sodium channel Nav1.7, which is extensively expressed in the peripheral (nociceptive and sympathetic fibers) as well as the central nervous system [68]. A wide range of pain-related disorders are associated with SCN9A mutations; primary erythromelalgia, characterized by episodes of intermittent burning pain and redness of extremities, was the first to be described and linked to missense mutations [69]. Shortly later, paroxysmal extreme pain disorder was associated with missense mutations, which prevent inactivation of Nav1.7 [70], while loss-of-function mutations were found in patients with congenital insensitivity to pain [71].

Subsequently, gain-of-function mutations were described in patients with SFN, also showing dorsal ganglion root hyperexcitability [72], and in patients with painful diabetic neuropathy [73].

SCN10A mutations, encoding Nav1.8, have been described in patients and families with painful and autonomic signs and symptoms [67,74], both as gain- and loss-of-function mutations.

SCN11 encodes Nav1.8, and mutations underlie hereditary sensory and autonomic neuropathy type 7 (HSAN7) [75]. SFN patients have also been found to present mutations of this gene [76].

Subsequently, there are hereditary disorders in which SFN can show somatic (e.g., Fabry disease) or autonomic (e.g., familial amyloidosis) predominance.

Fabry disease is caused by mutations of the α-galactosidase A (GLA) gene, which leads to multisystemic deposition of the glycolipid globotriaosylceramide. Pain and paresthesias affecting the extremities (acroparesthesias) are a predominant feature of the disease, occurring since childhood in classic forms of the disease. Extreme temperatures can exacerbate such symptoms. Involvement of the autonomic fibers of the gastrointestinal tract can lead to signs and symptoms that can be missed [77]. Painful signs and symptoms in Fabry disease are actually caused by a multifaceted set of pathophysiological mechanisms, first including dorsal root ganglion dysfunction, then small-fiber neuropathy, and central mechanisms [78].

Familial forms of amyloidosis, such as those caused by mutation of the transthyretin (TTR) gene, are characterized by autonomic SFN, as well as large-fiber involvement [67].

Ehlers–Danlos syndromes, a group of connective tissue disorders, are often characterized by pain, which was shown to be related to SFN [79].

### 4.6. Idiopathic

This is the most common etiology, but care must be taken not to overlook other potential causes of SFN. A key diagnostic aspect is differentiating such forms of SFN from other pain syndromes such as fibromyalgia, which in some subsets has shown alterations of intraepidermal innervation [80].

Relevant articles on the various etiologies of SFN are summarized in Table 3.

## 5. Treatment

Treatment of SFN should focus on the management of symptoms negatively affecting quality of life.

As indicated in Table 4, neuropathic pain can be treated by different classes of drugs, including gabapentinoids (pregabalin, gabapentin), serotonin and norepinephrine reuptake inhibitors (duloxetine, venlafaxine), and tricyclic antidepressants. Second-line approaches may include cannabinoids, topical capsaicin, and botulinum toxin type A (Table 4).

Other approaches, such as cognitive-behavioral therapy, are possible and complementary [82].

The role of the Nav1.7, Nav1.8, and Nav1.9 channels in painful syndromes has suggested that targeted therapy could provide treatment specific to neuropathic pain; antagonist molecules have the challenge of being specific enough to these sodium channels without impairing the fundamental functions of other voltage-gated sodium channels; other approaches such as gene therapy are promising but demanding [83].

Similarly, autonomic symptoms can be managed with lifestyle measures (e.g., chewing gum for sialorrhea) or with specific therapy if severe (e.g., fludrocortisone for orthostatic hypotension).

## 6. Conclusions

SFN is a vast group of different disorders impacting small nerve fibers in possibly different ways. In this narrative review, articles have been selected and presented with attention to etiologies; this was because SFN can occur in a wide variety of non-neurological disorders, and its recognition should be improved in both neurologists and non-neurologists.

The results of the article selection have shown several isolated case reports or series linking SFN with different causes. While in some instances data are scant, acknowledgment of unusual causes can prompt the collection of larger and stronger data, which should entail precise identification of SFN cases. This can be achieved via a thorough neurological examination, with special focus on signs of somatic and autonomic impairment of small nerve fibers, and diagnostic techniques such as skin biopsy, which offers the advantage of providing quantitative and reproducible data on innervation.

Furthermore, higher recognition of SFN in long-known causes is also needed. For several systemic disorders, such as diabetes, amyloidosis, and Fabry disease, SFN can be thought of as a prodromal form of neuropathy and can represent such a warning signal of disease activity [40], and its identification could thus lead to the early treatment and prevention of higher, more widespread damage.

In conclusion, recognizing the many different etiologies of SFN, both common and rare, can lead to better symptom management and quality of life for patients.

## Figures and Tables

**Table 1:** Classification of small nerve fibers.

Fiber Type	Conduction Velocity	Function	Receptors
Aδ: thinly myelinated	4–36 m/s	Cold sensation (<25 °C)	Transient Receptor Potential Cation Channel, Subfamily M, Member 8 (TRPM8)
		Heat sensation (>45 °C) and nociception	Transient Receptor Potential Cation Channel, Subfamily V, Member 1 and 2 (TRPV1 and TRPV2)
		Mechanical nociception	
C: unmyelinated	0.4–2 m/s	Warm sensation (>35 °C)	Transient Receptor Potential Cation Channel, Subfamily V, Member 3 (TRPV3)
		Cold sensation (<5 °C) and nociception	Transient Receptor Potential Cation Channel, Subfamily A, Member 1 (TRPA1), Subfamily V, Member 1 (TRPV1), and Subfamily M, Member 8 (TRPM8)
		Polymodal nociception	Transient Receptor Potential Cation Channel, Subfamily V, Member 1 (TRPV1) and Subfamily A, Member 1 (TRPA1)
		Postganglionic autonomic	Acetylcholine (parasympathetic)/Adrenergic receptors (sympathetic)

**Table 2:** Overview of diagnostic criteria for SFN.

Besta Criteria ≥2 of:	NEURODIAB Criteria	
(1) Clinical signs of small nerve fiber disease (2) Altered foot thermal threshold at quantitative sensory testing (3) Reduced intraepidermal nerve fiber density at distal leg as assessed by skin biopsy	(1) Length-dependent symptoms of small nerve fiber disease (2) Length-dependent signs of small nerve fiber disease (3) Normal sural nerve conduction studies (4) Reduced intraepidermal nerve fiber density at distal leg as assessed by skin biopsy (5) Altered foot thermal threshold at quantitative sensory testing	
	Possible SFN	1 and/or 2
	Probable SFN	1 and 2 and 3
	Definite SFN	1 and 2 and 3 and 4 and/or 5

**Table 3:** Literature review on SFN by etiology.

Paper	Summary
**Metabolic**	
Sumner et al., 2003 [40]	Explores the wide spectrum of diabetes-associated neuropathies
Pittenger et al., 2005 [42]	Patients with metabolic syndrome and pain may present reduced leg IENFD
Ørstavik et al., 2006 [43]	Altered QST in patients with hypothyroidism and painful symptoms
Güneş et al., 2018 [44]	Reduced IENFD in patients with vitamin B12 deficiency and pain
Chaudhry et al., 1999 [45]	28/58 (48%) of chronic liver disease patients presented with autonomic neuropathy
**Infectious**	
Boger et al., 2012 [46]	QSART may identify SFN in patients with HIV infection
Hashimoto et al., 2023 [50]	Reduced IENFD in patients with post-herpetic itch
Donadio et al., 2024 [53]	Somatic SFN in patients with SFN following COVID infection or vaccination
Falco et al., 2024 [54]	50% of patients with painful long COVID syndrome meet criteria for SFN
**Autoimmune**	
Oomatia et al., 2014 [57]	14/82 (17%) of SLE patients presented SFN, at times in non-length-dependent form
Liampas et al., 2023 [58]	Systematic review and meta-analysis of SFN in primary Sjögren syndrome
Gavrilova et al., 2021 [59]	Up to 60% of sarcoidosis patients present with a clinical picture congruent with SFN
**Drug-induced/Toxic**	
Giannoccaro et al., 2011 [63]	Bortezomib-associated SFN in three patients, as demonstrated by immunofluorescence
Krøigård et al., 2014 [64]	QST and skin biopsy disclose SFN in patients treated with oxaliplatin or docetaxel
Kokotis et al., 2023 [66]	8/16 (50%) of alcohol-dependent subjects presented with SFN (QST and skin biopsy)
**Inherited**	
Faber et al., 2012 [72]	Gain-of-function mutations of *SCN9A* can cause SFN
Dabby et al., 2016 [74]	Gain-of-function mutations of *SCN10A* can cause SFN
Eijkenboom et al., 2019 [76]	Potentially pathogenic mutations of voltage-gated sodium channels in 11.6% of SFN
Burand et al., 2021 [78]	Many different phenomena, including SFN, cause neuropathic pain in Fabry disease
Cazzato et al., 2016 [79]	SFN described in Ehlers–Danlos patients
**Other/Idiopathic**	
Giannoccaro et al., 2014 [80]	Skin biopsy in patients with fibromyalgia

**Table 4:** Lines of therapy for peripheral neuropathic pain according to the 2020 French guidelines (table derived from Figure 2 of [81]).

Therapy	Line	Notes
SNRIs (duloxetine, venlafaxine)	First	
Tricyclic antidepressants	First	
Gabapentinoids (gabapentin, pregabalin)	First (gabapentin), Second (pregabalin)	
Tramadol	Second	
Combination therapy (antidepressants + gabapentinoids)	Second	
Lidocaine plasters	First	Focal pain
TENS	First	Focal pain
Capsaicin patch	Second	Focal pain
Botulinum toxin A	Second	Focal pain
High-frequency repetitive transcranial magnetic stimulation	Third	
Spinal cord stimulation	Third	
Strong opioids	Third	

## Data Availability

Not applicable.
